# Right Diaphragm Spontaneous Rupture: A Surgical Approach

**DOI:** 10.1100/tsw.2011.91

**Published:** 2011-05-05

**Authors:** Duilio Divisi, Giovanna Imbriglio, Andrea De Vico, Roberto Crisci

**Affiliations:** Department of Thoracic Surgery, University of L'Aquila, “G. Mazzini” Hospital of Teramo, Italy

**Keywords:** spontaneous diaphragmatic lesion, radiological diagnosis, video-assisted thoracoscopy, surgical treatment

## Abstract

We present a case of spontaneous rupture of the diaphragm, characterized by nonspecific symptoms. The rapid diagnosis and appropriate surgical approach led to a positive resolution of the pathology.

## INTRODUCTION

"Spontaneous" describes a rupture of the diaphragm due to an increased pressure in the chest wall and abdominal cavity without direct trauma[1]. It is a rare and dangerous condition, often difficult to detect, and, for this reason, diagnosed late. A few cases of spontaneous diaphragmatic lesion associated with violent cough or vomiting are reported in patients with congenital or acquired defect of the muscle[2]. This study aims to describe the ideal diagnostic and therapeutic approach to this pathology.

## CASE REPORT

A 65-year-old man arrived at the emergency room complaining of a sudden acute thoracic and epigastric pain associated with progressive dyspnea that had arisen during the night. The pain was worse in the right side of the chest and extended to the back. The patient was in a forced semi-sitting position and showed shallow breathing and asthenia. He referred to incoercible vomiting and nausea in the last 72 h. There were no signs of bruises on his body. He did not remember any body traumas, he was well oriented, and was a heavy smoker (40 cigarettes/day). Blood gas analysis highlighted hypoxia ($PaO_2$: 54 mmHg), hypercapnia ($PaCO_2$: 50 mmHg), and an $O_2$ saturation rate of 80%. General blood exams demonstrated high values of transaminase, cholestasis markers, creatinine, and leukocytes. Increasing thoracic pain resistant to morphine and dyspnea counseled a chest X-ray that showed an opacity of two-thirds of the right hemithorax, with the absence of the right diaphragmatic line. The patient was transferred to the intensive care unit due to deterioration in respiratory parameters that required intubation and mechanical ventilation. Thin-section computerized tomography (CT) of the thorax and abdomen revealed a diaphragmatic herniation of the liver (Fig. 1). The patient underwent video-assisted thoracoscopy (VAT) 1.5 h following admission. The 8-mm trocar for the 0° optical camera was localized in the 5$^{th}$ intercostal space

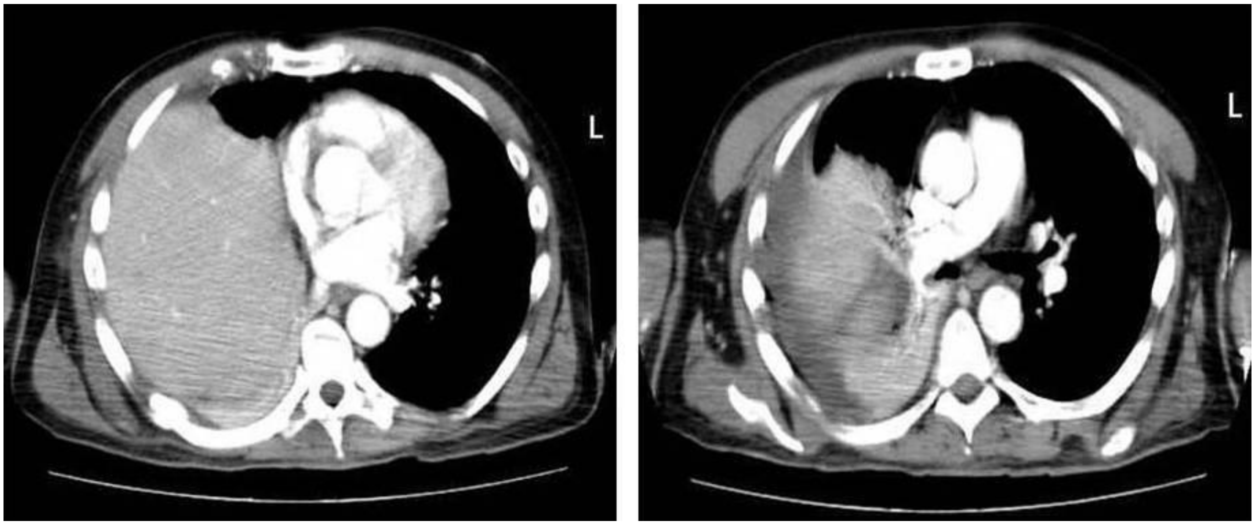

**FIGURE 1.** CT of the thorax highlights the obliteration of the pleural cavity by the liver and pulmonary parenchyma compression.

(I.S.) along the midaxillary line. VAT allowed clear evaluation of the pleural cavity, discovering a small serum-hematic effusion and a 10-cm rupture of the diaphragm in which was wedged the $7^{th}$ liver segment. Conversion to lateral thoracotomy was needed at the $6^{th}$ I.S. in order to dissect the liver and the pulmonary adhesions (Fig. 2), to replace the liver in the abdominal cavity, and to reduce its ischemia time. The diaphragmatic lesion was repaired by suture with interrupted polyfilament stitches (Fig. 3). Two pleural drainages n° 28 Ch and n° 36 Ch were placed at the end of the intervention. The respiratory parameters improved quickly, and the patient was extubated and transferred to the thoracic surgery unit 48 h after surgery. Pleural drainages were removed during the $5^{th}$ and $7^{th}$ day after the surgical procedure, and the patient was discharged from the hospital on the $8^{th}$ postoperative day. The follow-up at 12 months with chest X-ray and CT of the thorax showed the strength of the diaphragmatic suture, the lung re-expansion, and the correct placement of the liver (Fig. 4).

## DISCUSSION

Diaphragmatic rupture is due to a penetrating or closed trauma of the thorax and abdomen. It was described for the first time by Sennertus in the $16^{th}$ century, while the first two deaths were described by Ambrose Paré in 1578, one deriving from a strangulated bowel[3]. Only in the second half of $20^{th}$ century was the spontaneous etiology in the rupture of the diaphragm considered as a consequence of physical effort[4], delivery[5,6], or violent cough[7]. The sudden increase in abdominal pressure and the transdiaphragmatic gradient explain the nonspecific symptoms and diagnostic difficulty[8]. The particularity of our experience is the rupture in the right side of the diaphragm. In fact, lesions are usually placed in the left diaphragmatic side, which is not protected by the liver. Our clinical considerations were (1) the decline in the general condition of the patient, with a muscle hypotrophy and hypotonicity, and the slackness of the diaphragm; (2) a large and cirrhotic liver; and (3) a particular strain due to vomiting. Thoracotomy was preferred to laparotomy owing to the symptoms and the CT evaluation that showed a dislocation of the liver in the pleural cavity without the abdominal lesion. We think that in cases of easy and safe dissection, the main technique is the suture with separate stitches. It is best to avoid sutures with uninterrupted stitches because it is not unusual to have recurrence. Mesh is indicated when it is impossible to restore diaphragmatic continuity by suture owing to fibrosis and inelastic tissue. The laparotomic approach could have been dangerous and more difficult than thoracotomy in the presence of

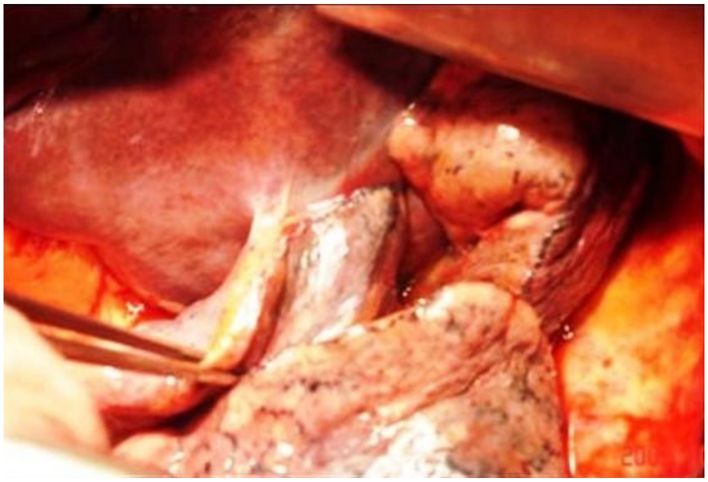

**FIGURE 2.** Liver herniation in the thorax with collapse of middle and lower lobes.

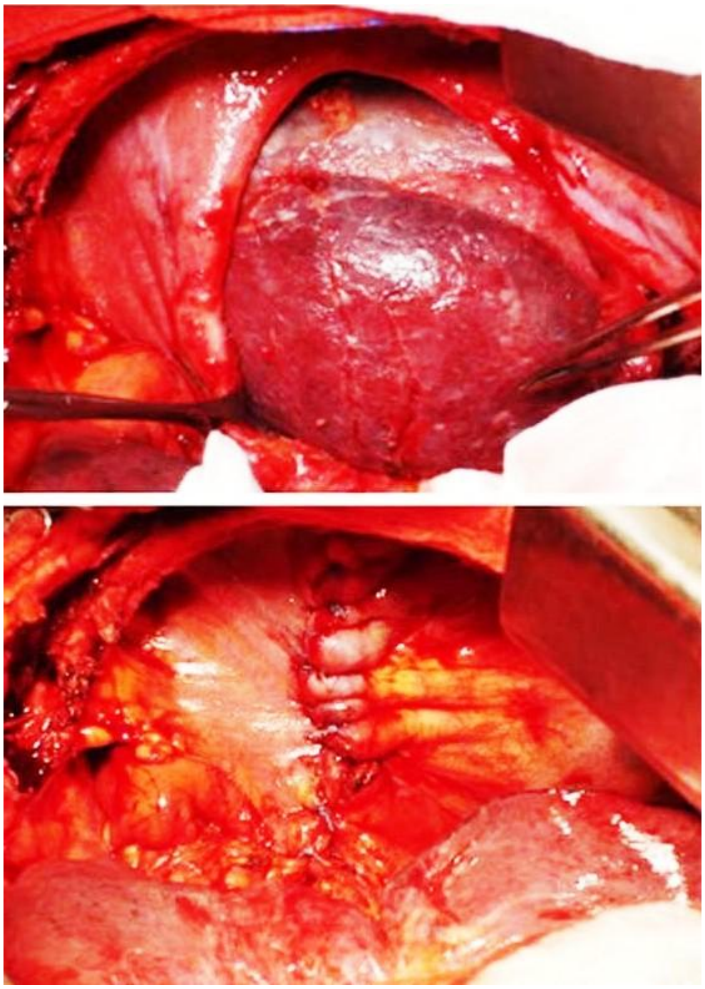

**FIGURE 3.** The liver reduction in the abdomen and diaphragmatic suture with separate stitches.

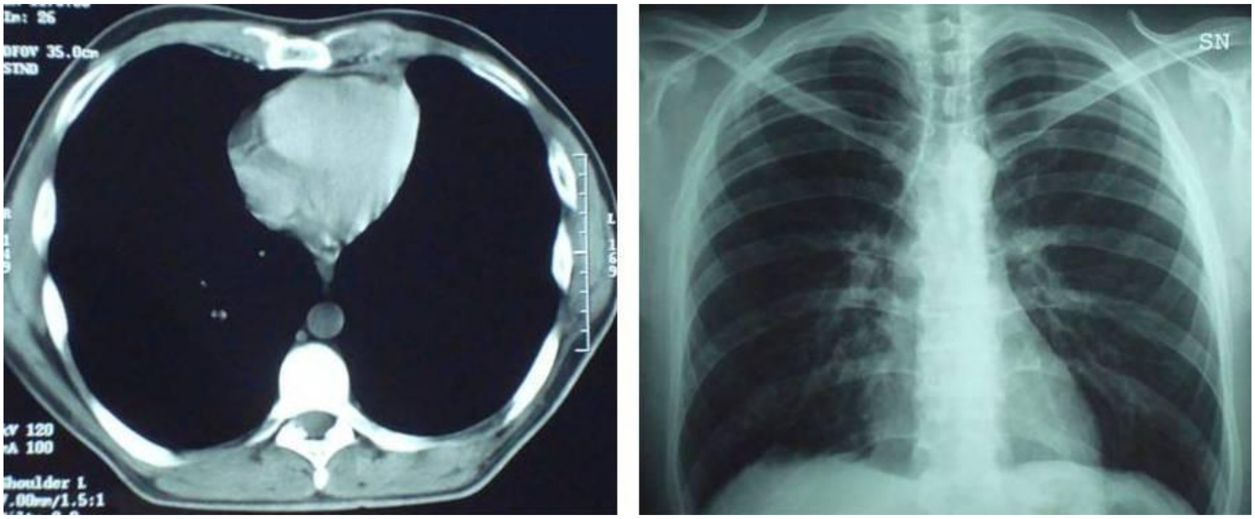

**FIGURE 4.** CT of the thorax and chest X-ray at 12 months showed maintenance of a normal anatomical position of the lung as well as the diaphragmatic line.

adhesions between the dislocated bowel and pleura. In our study, thoracoscopy was used for diagnosis. The wide adhesions between the liver and middle and inferior pulmonary veins advise against the VAT conversion to VATS.

In an experienced thoracic surgery unit, bigger ruptures can also be treated with VATS.

## CONCLUSIONS

The most dangerous complication of a diaphragmatic rupture, both spontaneous and traumatic, is the dislocation of the abdominal bowels into the chest cavity and their ischemia. The chest X-ray orientates the diagnosis that must be confirmed in emergency situations with CT multislices and VAT. Magnetic resonance imaging of the thorax and abdomen is best used in elective situations, as well as abdominal ultrasound in a low-risk setting. There is no single and standardized surgical treatment, but it depends rather on the time elapsed between the acute event and hospitalization. The worsening of symptoms and complications are more common in a lesion late diagnosed, as a consequence of fibrosis between dislocated bowel and pulmonary parenchyma. Laparotomy does not allow easy replacement of dislocated bowels that could be necrotic. Thoracotomy is the elective surgical approach that ensures the correct anatomical restoration of the chest and abdominal cavity by separate suture stitches.
